# Modelling the long-term benefits of tolvaptan therapy on renal function decline in autosomal dominant polycystic kidney disease: an exploratory analysis using the ADPKD outcomes model

**DOI:** 10.1186/s12882-019-1290-5

**Published:** 2019-04-23

**Authors:** Hayley Bennett, Phil McEwan, Karina Hamilton, Karl O’Reilly

**Affiliations:** 1 Health Economics and Outcomes Research Ltd, Cardiff, UK; 2 Swansea Centre for Health Economics, Swansea University, Swansea, UK; 3 Otsuka Pharmaceutical Europe Ltd, Gallions Wexham Springs, Framewood Road, Wexham, SL3 6PJ UK

**Keywords:** Autosomal dominant polycystic kidney disease, Disease modelling, Tolvaptan, End-stage renal disease, Renal function decline

## Abstract

**Background:**

The short-term efficacy of tolvaptan in patients with autosomal dominant polycystic kidney disease (ADPKD) has been demonstrated across several phase 3 trials, while the ADPKD Outcomes Model (ADPKD-OM) represents a validated approach to predict natural disease progression over a lifetime horizon. This study describes the implementation of a tolvaptan treatment effect within the ADPKD-OM and explores the potential long-term benefits of tolvaptan therapy in ADPKD.

**Methods:**

The effect of tolvaptan on ADPKD progression was modelled by applying a constant treatment effect to the rate of renal function decline, consistent with that observed in the Tolvaptan Efficacy and Safety in Management of Autosomal Dominant Polycystic Kidney Disease and Its Outcomes trial (TEMPO 3:4; ClinicalTrials.gov identifier NCT00428948). Predictions generated by the ADPKD-OM were compared against aggregated data from a subsequent extension trial (TEMPO 4:4; ClinicalTrials.gov identifier NCT01214421) and the Replicating Evidence of Preserved Renal Function an Investigation of Tolvaptan Safety Efficacy in ADPKD trial (REPRISE; ClinicalTrials.gov identifier NCT02160145). Following validation, an application of the ADPKD-OM sought to estimate the benefit of tolvaptan therapy on time to end-stage renal disease (ESRD), in a range of ADPKD populations.

**Results:**

Model validation against TEMPO 4:4 and REPRISE demonstrated the accuracy and generalisability of the tolvaptan treatment effect applied within the ADPKD-OM. In simulated patients matched to the overall TEMPO 3:4 trial population at baseline, tolvaptan therapy was predicted to delay the mean age of ESRD onset by five years, compared to natural disease progression (57 years versus 52 years, respectively). In subgroup and sensitivity analyses, the estimated delay to ESRD was greatest among patients with CKD stage 1 at baseline (6.6 years), compared to CKD 2 and 3 subgroups (4.7 and 2.7 years, respectively); and ADPKD patients in Mayo subclasses 1C–1E.

**Conclusions:**

This study demonstrated the potential for tolvaptan therapy to delay time to ESRD, particularly among patients with early-stage CKD and evidence of rapidly progressing disease. Data arising from this study highlight the value to be gained by early intervention and long-term treatment with tolvaptan, which may alleviate the economic and societal costs of providing care to patients who progress to ESRD.

## Background

Autosomal dominant polycystic kidney disease (ADPKD) is the most common inherited renal disease, characterised by the progressive deterioration of normal kidney tissue, development of chronic kidney disease (CKD), and the onset of end-stage renal disease (ESRD) [1]. The burden of ADPKD on patients and healthcare systems is largely driven by its incurable progression towards ESRD, and the subsequent requirement for renal replacement therapy (RRT). Patients with ADPKD account for approximately 10% of those dependent on RRT in Europe; and the 2010 cost of providing RRT to ADPKD patients across the European Union (EU) was estimated at €1.5 billion [2].

In 2014, tolvaptan, a selective vasopressin V_2_ antagonist, became the first pharmacological agent to receive regulatory approval for the management of ADPKD. Since its initial approval in Japan, tolvaptan has subsequently received marketing authorisation for the treatment of adult ADPKD patients in the EU, Canada, South Korea, Switzerland, Hong Kong, Australia, and most recently, the United States [3–7]. Regulatory approvals of tolvaptan in ADPKD were primarily informed by the phase 3 Tolvaptan Efficacy and Safety in Management of Autosomal Dominant Polycystic Kidney Disease and Its Outcomes trial (TEMPO 3:4; ClinicalTrials.gov identifier NCT00428948), which compared tolvaptan against placebo in patients with early ADPKD and evidence of rapidly progressing disease [8]. Over the course of 3 years, TEMPO 3:4 found that tolvaptan therapy slowed the growth of total kidney volume (TKV), delayed renal function decline, and reduced the incidence of ADPKD-related kidney pain, haematuria and urinary tract infection [8, 9]. When the efficacy endpoints of TEMPO 3:4 were re-assessed in post hoc analyses, the observed clinical benefits of tolvaptan were found to be similar among patients across CKD stages 1–3 [10]. In a subsequent two-year, open-label extension trial (TEMPO 4:4; ClinicalTrials.gov identifier NCT01214421), sustained protection of renal function was evidenced in the TEMPO 3:4 treatment arm over 5 years of tolvaptan therapy [11]. Recently, the Replicating Evidence of Preserved Renal Function an Investigation of Tolvaptan Safety Efficacy in ADPKD trial (REPRISE; ClinicalTrials.gov identifier NCT02160145) aimed to assess the benefits of tolvaptan in later-stage ADPKD (CKD stages 2–4), and showed that treatment was associated with a slower decline in estimated glomerular filtration rate (eGFR) over 1 year, compared to placebo [12].

Clinical trials to-date have collectively demonstrated the safety and efficacy of tolvaptan therapy for up to 5 years, in ADPKD patients across all CKD stages. The longer-term benefit of tolvaptan therapy is uncertain, but may be estimated utilising clinical prediction modelling strategies such as computer-based patient simulations. Previously, data from the placebo arm of the TEMPO 3:4 trial was used to develop the ADPKD Outcomes Model (ADPKD-OM) [13]; a validated natural history model of ADPKD, which can be used to simulate progression of TKV and renal function across a range of ADPKD patient profiles and estimate the incidence of long-term clinical outcomes important to both clinicians and patients, such as time to ESRD.

The objectives of this study were to incorporate a treatment effect within the ADPKD-OM using available clinical trial data and to estimate the benefit of long-term tolvaptan therapy on ESRD onset in ADPKD patients.

## Methods

### Modelling the natural history of ADPKD

**Fig. 1:**
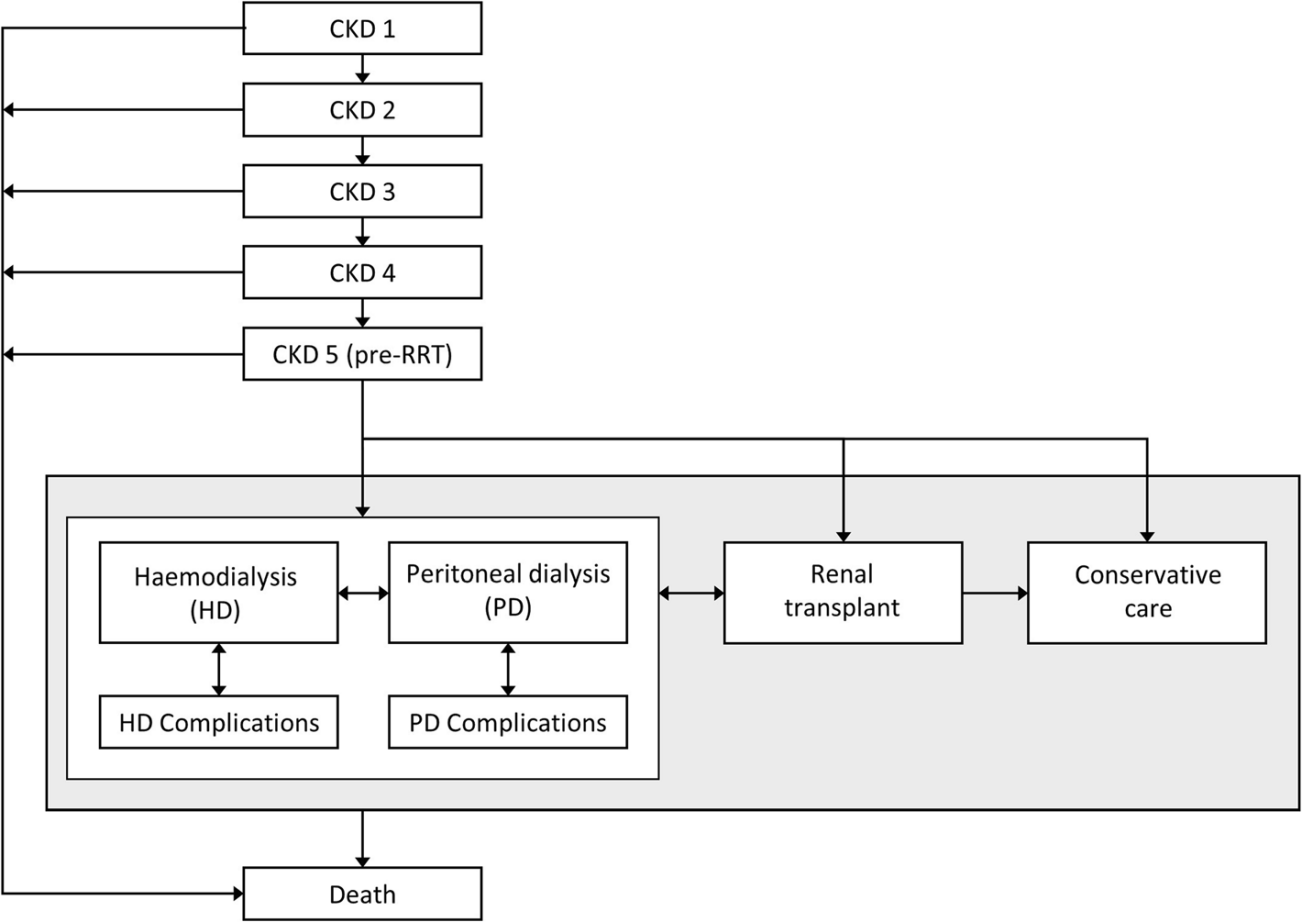
Flow diagram of health states modelled in the ADPKD Outcomes Model. Following the initiation RRT (grey box), patients were modelled across conservative care, dialysis and transplant health states

As described in the model publication [13], the ADPKD-OM is a patient-level simulation designed to predict the natural history of ADPKD based on readily available and/or measurable clinical characteristics. Non-linear trajectories of TKV and eGFR are predicted for untreated patients as a function of age, sex, TKV and eGFR at baseline, according to the relationships seen between these variables in the placebo arm of TEMPO 3:4. Patients are modelled over a lifetime horizon, as shown in Fig. 1; progression through CKD stages is controlled by the eGFR trajectories, while published rates control the management of ESRD (via conservative care, dialysis or transplantation).

### Modelling the benefit of tolvaptan on ADPKD progression

The effect of tolvaptan therapy on ADPKD progression was added to the ADPKD-OM by applying a constant reduction to the rate of renal function (eGFR) decline predicted for an untreated patient. Consistent with TEMPO 3:4 observations, the natural rate of eGFR decline was reduced by 31.6% when measured using the reciprocal of serum creatinine, or 26.4% when using the Chronic Kidney Disease Epidemiology Collaboration (CKD-EPI) equation [14].

### Model validation

The modelled effect of tolvaptan on eGFR decline was validated against five-year observations from patients who entered TEMPO 4:4 from the TEMPO 3:4 treatment arm (defined as the early-treated cohort) [11] and one-year observations from CKD 2–4 patients in REPRISE [12, 15]. Simulated patient characteristics were matched to the early-treated cohort at TEMPO 3:4 baseline and to the REPRISE trial population at baseline, respectively (Table 1). The effect of continuous tolvaptan therapy was modelled as a 26.4% reduction in annual eGFR decline (measured by CKD-Epi). Modelled eGFR decline was compared with observed data from each trial.

**Table 1:** Patient characteristics of the TEMPO 3:4, TEMPO 4:4 and REPRISE trial populations for use in model validation exercises and illustrative analyses

Trial population	Cohort size (n)	Baseline characteristics, mean (SE)^a^				Source
		Age (years)	Female (%)	eGFR (mL/min/1. 73m^2^)	TKV (mL)	
Model application						
TEMPO 3:4 overall cohort	1445	38.7	48.4	81.61	1692.30	[34]
TEMPO 3:4 subgroups:						
- CKD 1	502	34.3	52.7	105.69	1353.76	[10]
- CKD 2	689	40.3	49.5	75.00	1712.00	[10]
- CKD 3	248	42.0	36.7	51.34	2323.18	[10]
- CKD 1–3; Mayo subclasses 1C-1E	1285	38.0	46.1	81.29	1760.40	[35]; Otsuka data on file
Model validation						
TEMPO 4:4 early-treated cohort^b^	557	38.95 (0.29)^c^	45.60 (2.11)	81.35 (0.68)^d^	1706.00 (37.35)	[11, 36]
REPRISE overall cohort	1370	47.50 (0.22)	50.40 (0.01)	41.00 (0.30)	2026.30 (37.88)	[12, 15]

*CKD* chronic kidney disease, *eGFR* estimated glomerular filtration rate, *SE* standard error, *TKV* total kidney volume

^a^SE derived from standard deviations and patient numbers reported in the original trial publications

^b^Reported values reflect the characteristics of the TEMPO 4:4 early-treated cohort at TEMPO 3:4 baseline

^c^Age of patients at TEMPO 3:4 baseline was assumed to be 3.25 years less than the reported baseline age in TEMPO 4:4, based on the duration of TEMPO 3:4 (3 years) and the off-treatment period between TEMPO 3:4 and TEMPO 4.4 (approximately 3 months)

^d^Baseline eGFR was not reported in TEMPO 4:4; thus, it was assumed that baseline eGFR among the matched population was equal to the full TEMPO 3:4 population [8]

### Model application

Following validation, the ADPKD-OM was used to estimate the potential long-term renal benefits of tolvaptan therapy in hypothetical ADPKD cohorts. Modelled patients were given baseline characteristics consistent with the mean profiles of the overall TEMPO 3:4 trial population, or subgroups defined by CKD stage (Table 1), and the effect of tolvaptan was modelled as a 31.6% reduction in annual eGFR decline (measured using the reciprocal of serum creatinine). For each modelled population, predicted ADPKD progression in the tolvaptan treatment arm was compared against natural ADPKD progression (control arm). The potential delay to ESRD associated with treatment was assessed, assuming no discontinuation of tolvaptan therapy prior to ESRD onset.

### Sensitivity and scenario analyses

To assess their influence on model predictions of ADPKD progression, model inputs related to treatment discontinuation, ADPKD progression within the trial period, and the effect of tolvaptan on eGFR based on the CKD-Epi equation were modified in sensitivity analysis. Additional scenario analysis estimated the impact of tolvaptan therapy on time to ESRD for patients stratified by CKD stage at initiation, and in ADPKD subclasses 1C-1E using the Mayo imaging classification system [16]. Modified inputs applied in sensitivity and scenario analyses are provided in Table 2; baseline patient characteristics applied in each are reported in Table 1.

**Table 2:** Model inputs modified in sensitivity and scenario analyses

Modified variables			Modified inputs
Sensitivity analysis			
Discontinuation of treatment^a,b^			15.30, 6.51, 2.89 and 0.50% in years 1, 2, 3 and 4+, respectively
Replication of ADPKD progression observed in TEMPO 3:4^a^			Percentage change in TKV from baseline with placebo: 5.05, 11.49 and 18.85% in years 1, 2 and 3, respectively Annual eGFR slope (mL/min/1. 73m^2^): − 3.812 with placebo − 2.609 with tolvaptan
Treatment effect based on CKD-Epi equation^a^			26.4% reduction in eGFR decline
Scenario analysis			
Treatment effect by CKD stage at tolvaptan initiation^a^		CKD 1	14.7% reduction in eGFR decline
		CKD 2	31.0% reduction in eGFR decline
		CKD 3	40.5% reduction in eGFR decline
Treatment effect based on CKD-Epi equation	CKD stage at tolvaptan initiation^c^	CKD 1	15.5% reduction in eGFR decline
		CKD 2	29.1% reduction in eGFR decline
		CKD 3	31.0% reduction in eGFR decline
	Mayo imaging classification at tolvaptan initiation^d^	1C-1E	28.2% reduction in eGFR decline

*CKD* Chronic kidney disease, *CKD-Epi* Chronic Kidney Disease Epidemiology Collaboration, *eGFR* estimated glomerular filtration rate, *TKV* total kidney volume

^a^Observed in TEMPO 3:4 [8, 34]

^b^Rate extrapolated after year 3

^c^Treatment effects reported for CKD 1 to CKD 3 subgroups in TEMPO 3:4 [10]; eGFR was estimated using the CKD-Epi equation [14]

^d^Treatment effect (eGFR slope − 2.82 mL/min/1. 73 m2 for tolvaptan versus − 3.93 mL/min/1. 73 m2 in placebo) reported for Mayo subclass 1C-E patients in TEMPO 3:4 [35]; eGFR was estimated using the CKD-Epi equation [14]

## Results

### Model validation

**Fig. 2:**
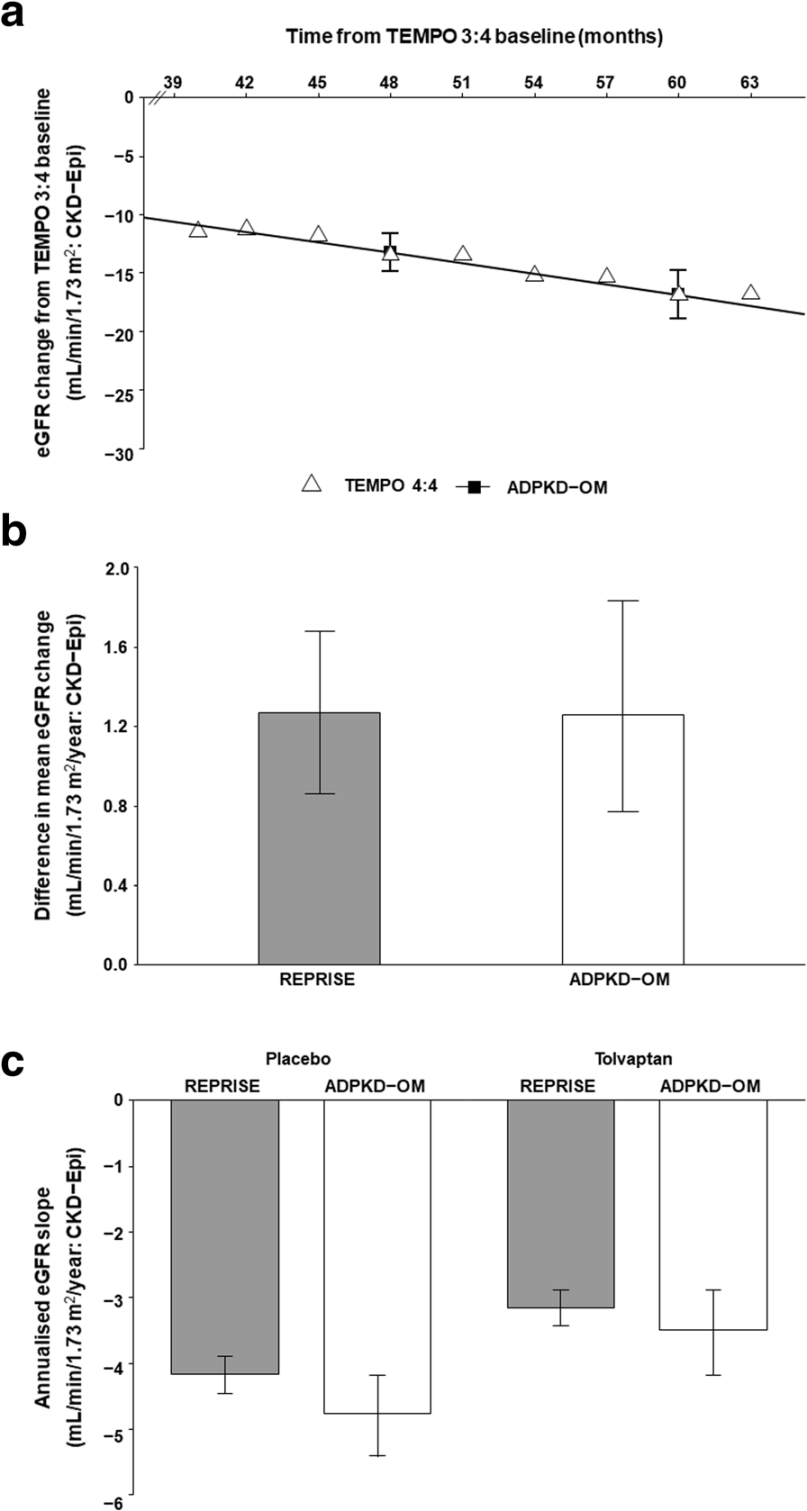
Validation of tolvaptan treatment effect applied in the ADPKD Outcomes Model. **a** Least squares mean eGFR change as observed in the early-treated cohort of TEMPO 4:4, assuming an average washout period of 3 months between TEMPO 3:4 and TEMPO 4:4 (unfilled triangles), compared against ADPKD-OM predictions (black filled squares joined by line). **b** Effect of tolvaptan on one-year eGFR change, as observed in REPRISE (grey filled bars) and predicted by the ADPKD-OM (unfilled bars). **c** Annualised eGFR slopes for tolvaptan and placebo arms, as observed in REPRISE (grey filled bars) and predicted by the ADPKD-OM (unfilled bars). Error bars indicate 95% confidence intervals; eGFR was estimated using the CKD-Epi equation

For simulated patients with characteristics matched to the early-treated TEMPO 4:4 cohort, ADPKD-OM predictions of five-year eGFR decline were consistent with data collected during the two-year extension period of the TEMPO 4:4 study (Fig. 2a). When simulated CKD 2–4 patients matched to the overall REPRISE population were modelled, the effect of tolvaptan therapy on mean eGFR change was predicted to be 1.26 mL/min/1. 73m^2^/year (95% confidence interval: 0.77, 1.83), compared to the trial primary endpoint of 1.27 mL/min/1. 73m^2^/year (0.86, 1.68) (Fig. 2b). Similarly, predicted eGFR slopes for cohorts simulated with and without treatment were consistent with those observed in the tolvaptan and placebo arms of the REPRISE study, respectively (secondary endpoint) (Fig. 2c).

### Model application

**Fig. 3:**
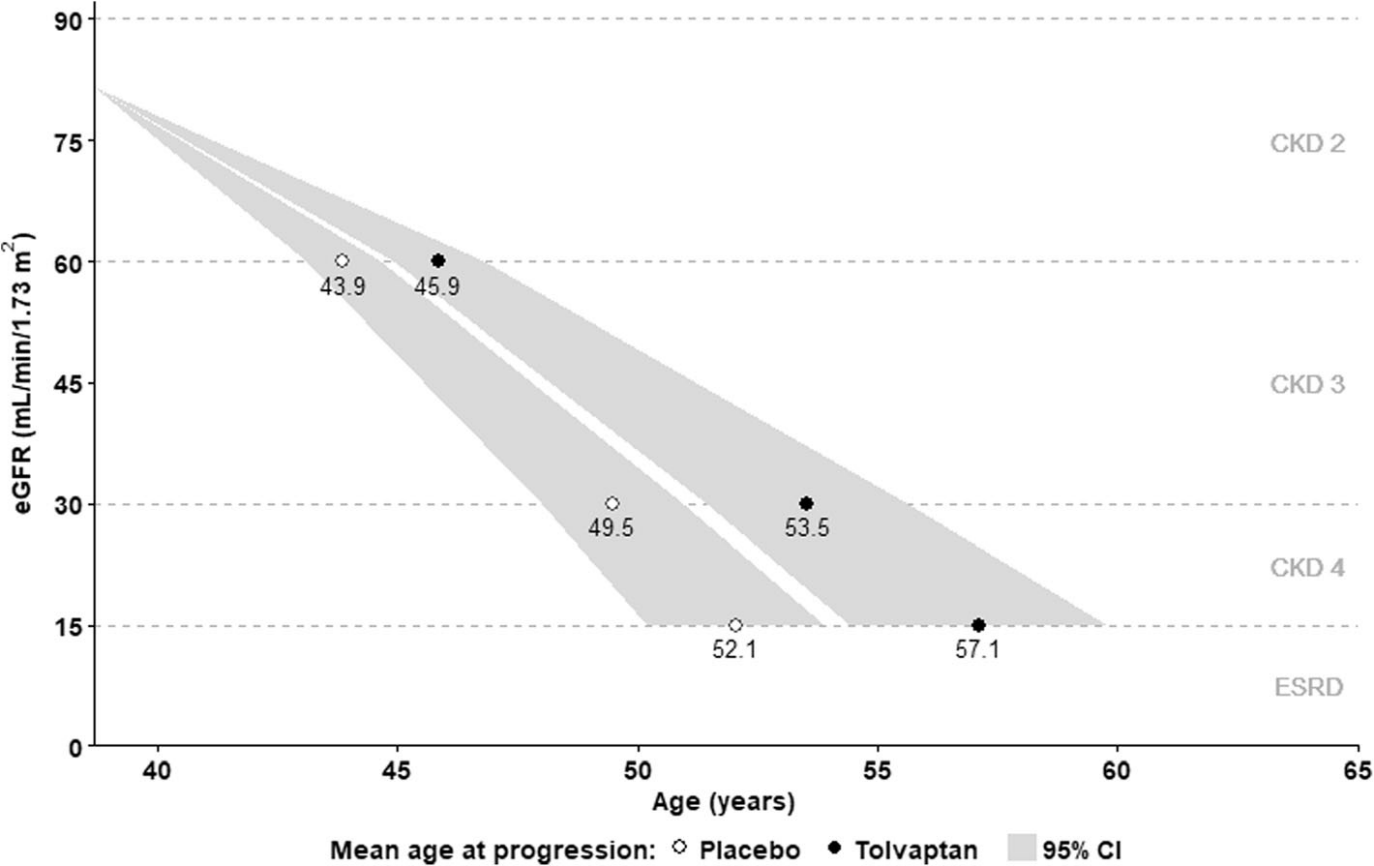
Predicted trajectory of CKD progression in modelled ADPKD patients treated with tolvaptan (black filled points), compared to natural history (white filled points). Labelled points indicate mean values; shaded regions indicate 95% confidence intervals; eGFR was estimated using the reciprocal of serum creatinine level

**Fig. 4:**
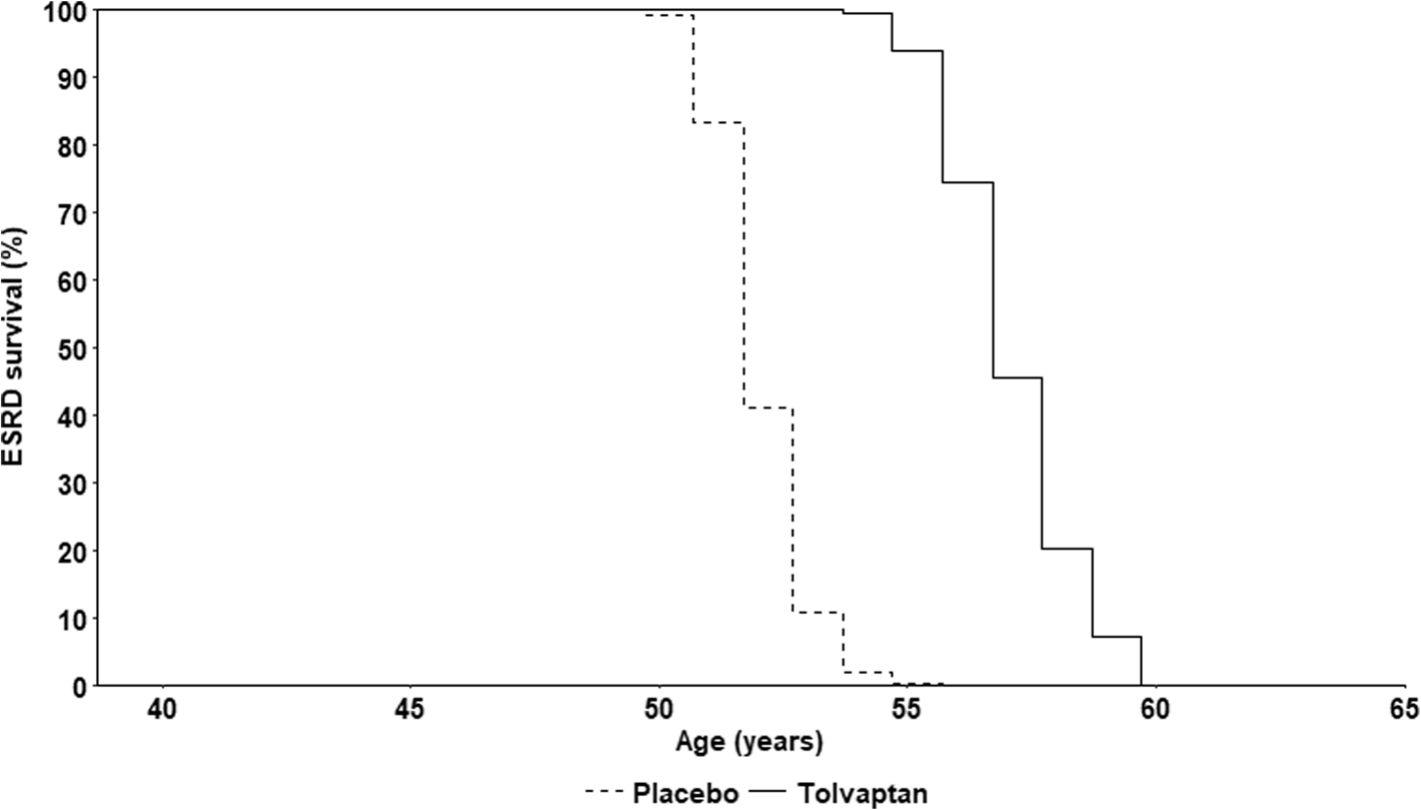
Kaplan-Meier plot of predicted survival to ESRD in modelled ADPKD patients treated with tolvaptan (solid line), compared to natural history (dashed line)

The predicted effect of tolvaptan on progression through CKD stages to the onset of ESRD among patients matched to the overall TEMPO 3:4 trial population are shown in Figs. 3 and 4, respectively. Without treatment, 96% of patients were estimated to reach ESRD prior to death; the modelled onset of ESRD occurred approximately 13 years from baseline, at a mean age of 52 years. In comparison, tolvaptan therapy was predicted to prevent ESRD in 3% of the simulated cohort, and delay ESRD onset by 5.1 years. The mean duration of treatment was 17.9 years; thus, on average, predicted ESRD onset was delayed by one year for every 3.5 years of tolvaptan therapy.

In modelled subgroups with CKD stage 1, 2 and 3 at baseline, the mean predicted time to ESRD under natural history was approximately 19, 12 and 7 years, respectively. Tolvaptan therapy delayed ESRD onset by 6.6 years among patients with CKD stage 1 at baseline, compared with 4.7 years and 2.7 years in CKD 2 and CKD 3 subgroups, respectively.

### Sensitivity and scenario analyses

**Fig. 5:**
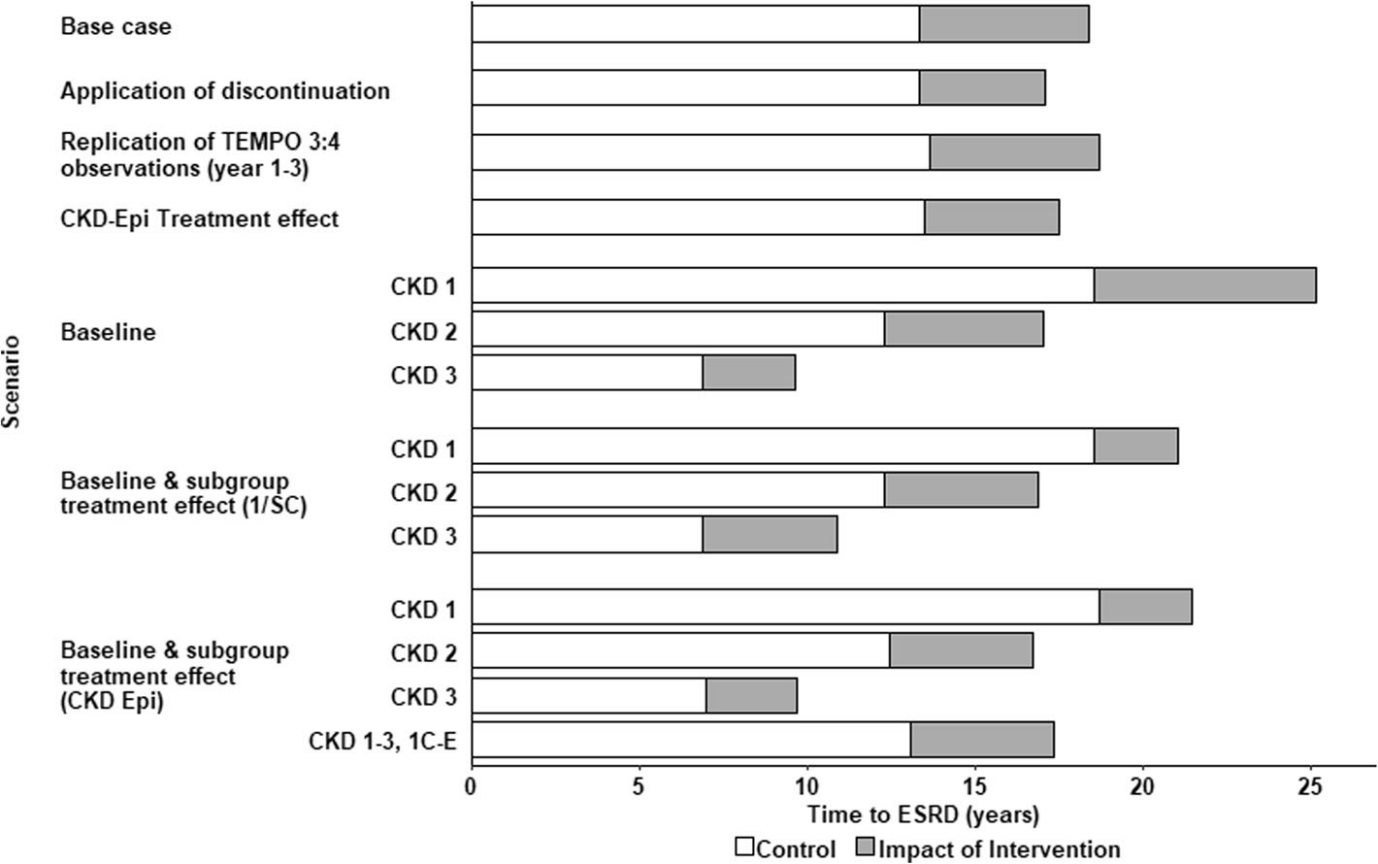
Influence of modelled scenarios on predicted time to ESRD, relative to the base case analysis in which the treatment effect measured by reciprocal of serum creatinine was applied to the overall TEMPO 3:4 population, without discontinuation. White bars indicate model predictions under natural history; grey bars indicate the incremental effect of tolvaptan on predicted time to ESRD

Model predictions of time to ESRD were sensitive to variations in baseline patient characteristics, the modelled duration of treatment, and the treatment effect applied (Fig. 5). Relative to the base case, applying treatment discontinuation rates observed in TEMPO 3:4 reduced the mean duration of treatment to 13.6 years, and the predicted delay to ESRD to 3.8 years. However, this equates to a one-year delay in ESRD onset for every 3.6 years of tolvaptan therapy, which is similar to that predicted in the base case.

Compared to the reciprocal of serum creatinine level used to measure eGFR in the base case analysis, modelled treatment effects based on the CKD-Epi equation typically reduced the estimated impact of treatment on ADPKD progression; however, meaningful delays to ESRD were nevertheless predicted. When the effect of tolvaptan on eGFR decline was modelled in patients restricted to Mayo subclasses 1C–1E, the ADPKD-OM predicted an additional delay to ESRD of approximately one-third of a year, when eGFR was measured by CKD-Epi (4.3 years versus 4.0 years).

## Discussion

The renoprotective benefits of tolvaptan for ADPKD management have been evidenced in several phase 3 trials, with TEMPO 3:4, TEMPO 4:4 and REPRISE collectively demonstrating its safety and efficacy for up to 5 years of treatment [8, 11, 12]. This study incorporated a treatment effect within the ADPKD-OM to estimate the potential benefits of longer-term tolvaptan therapy beyond the trial horizon; in particular, delayed onset of ESRD as a result of slowed renal function decline.

In this study, the modelled effect of tolvaptan therapy based on TEMPO 3:4 was compared to observed TEMPO 4:4 data, which demonstrated the accuracy with which the ADPKD-OM extrapolated the benefit of tolvaptan on eGFR decline over 5 years. Additional validation against REPRISE supported the generalisability of model predictions to patients with later-stage ADPKD, while subgroup analyses found that the predicted delay to ESRD in treated CKD 3 patients (2.7 years) was consistent with reported extrapolations of REPRISE data [12]. Furthermore, predictions generated by the ADPKD-OM were deemed sufficiently robust to support health technology assessments of tolvaptan by European regulatory agencies, including the National Institute for Health and Care Excellence (NICE) and Scottish Medicines Consortium (SMC) [17, 18].

In the presented application of the ADPKD-OM, tolvaptan therapy was predicted to delay ESRD onset by approximately 5 years in patients matched to the overall TEMPO 3:4 trial population. Despite differences in modelling methodologies and assumptions, this finding is broadly comparable with a previous cost-effectiveness analysis of tolvaptan, which estimated a 6.5-year reduction in the median age at ESRD onset [19]. Other extrapolations of TEMPO 3:4 data have estimated that every 4 years of tolvaptan therapy would delay the incidence of ESRD by 1 year [3]; a similar result was observed in our base case scenario, and in sensitivity analysis where treatment discontinuation was applied. In additional analyses, the predicted benefits of tolvaptan on time to ESRD were greater in patients with early-stage disease, particularly those with CKD stage 1 at baseline. Greater delays to ESRD were also predicted among patients in Mayo subclasses 1C-1E; a population previously identified in European Renal Association – European Dialysis and Transplant Association (ERA-EDTA) treatment algorithms as susceptible to rapid disease progression, and thus more likely to benefit from tolvaptan therapy [3].

The predicted effect of tolvaptan on time to ESRD is noteworthy, given that the public health burden of ADPKD is driven by the high costs and low utility associated with RRT. The incurable progression of ADPKD is the leading inheritable cause of ESRD, the management of which has been associated with annual per-patient costs ranging between €30,000 and €60,000 across European settings [20–23]. In addition to economic costs, the requirement for RRT also carries a heavy societal burden, due to increased provision of disability pensions and sickness allowances [24], its detrimental effects on patient productivity and quality of life [25–27], and a survival prognosis comparable to that of colorectal and lung cancers [28]. As the health economic impact of ADPKD intensifies with its progression towards ESRD, the predictions generated in this study highlight the potential for timely and long-term tolvaptan therapy to improve outcomes for patients and healthcare systems.

The modelling approaches adopted in this study are associated with some limitations. Although TEMPO 3:4 represents one of the largest, high-quality data sources in the field of ADPKD, its enriched trial design led to the inclusion of a relatively homogeneous cross-section of the ADPKD population and as such caution is required when generalising ADPKD-OM predictions to alternative patient populations, particularly those without early-stage and rapidly progressing disease. There is a high degree of variability associated with the rate of ADPKD progression [29]; though a linear decline in eGFR is observed in many ADPKD patients reaching ESRD, more complex patterns of eGFR with deviations from linearity (unrelated to acute events) are not uncommon [30, 31], which emphasises the utility of methods to accurately predict longer term progression for a patient cohort and potentially inform patient management. Validation exercises performed in this study were limited by a variable washout period between the TEMPO trials and a paucity of TKV data in REPRISE; despite this, predictions generated by the ADPKD remained consistent with observed data. The effect of tolvaptan therapy on ADPKD progression was modelled by applying a constant treatment effect to the rate of eGFR decline predicted for an untreated patient, despite trial evidence suggesting greater renal benefits among patients with later-stage CKD [8, 10]. Our analyses did not capture other reported benefits of tolvaptan therapy, including reductions in TKV growth and serious kidney pain events [8, 9]. Hypertension is a significant independent risk factor for progression to ESRD in patients with ADPKD [32] and achievement of low systolic blood pressure in early ADPKD has been associated with a slower loss of renal function [33], however blood pressure was not considered in our study. Furthermore, the ADPKD-OM did also not account for proteinuria or extra-renal complications such as polycystic liver and cardiovascular diseases. These conservative approaches are likely to underestimate both the true health economic burden of ADPKD, and the subsequent clinical value of tolvaptan therapy for ADPKD management. Finally, our base case analysis explored the benefit of continuous tolvaptan therapy and assumed no treatment discontinuation between initiation and ESRD onset; however, this parameter was shown to be influential in sensitivity analysis. Due to its aquaretic mechanism of action, tolvaptan has been associated with increased thirst, polyuria and nocturia [8]; however, ERA-EDTA, European Medicines Agency and NICE guidance state that such adverse effects are generally adaptable, and that successful drug adherence is achievable with increased patient screening, education and monitoring [3, 5, 17]. These recommendations were informed by TEMPO 3:4, which found that the majority of patients who discontinued tolvaptan therapy due to an aquaresis-related adverse event (8.3%) did so during the first month of treatment [8].

## Conclusions

The burden of ADPKD on patients and healthcare systems is largely driven by its progression towards ESRD; however, clinical trials are unlikely to capture the long-term value of therapeutic strategies aimed to delay disease progression. Using available trial data, this study implemented and validated a tolvaptan treatment effect within the ADPKD-OM, in order to predict the effect of therapy on clinical outcomes over a lifetime horizon. Relative to natural ADPKD progression, tolvaptan was associated with noteworthy delays to ESRD onset among modelled ADPKD cohorts, particularly those with early-stage and rapidly progressing disease. Despite limitations with regards to the generalization of the results to all ADPKD patients, the data from this study highlights the potential value of early intervention and long-term treatment with tolvaptan, which may alleviate the economic and societal costs of providing care to ADPKD patients who progress to ESRD. As tolvaptan therapy becomes increasingly available to ADPKD patients worldwide, its predicted benefits have great potential to be realised.

## Abbreviations

ADPKD: Autosomal dominant polycystic kidney disease
ADPKD-OM: Autosomal dominant polycystic kidney disease outcomes model
CKD: Chronic kidney disease
CKD-Epi: Chronic Kidney Disease Epidemiology Collaboration
CRISP: Consortium for radiologic imaging studies of polycystic kidney disease study
eGFR: Estimated glomerular filtration rate
ERA-EDTA: European Renal Association-European Dialysis and Transplantation Association
ESRD: End-stage renal disease
EU: European Union
HALT-PKD: Halt Progression of Polycystic Kidney Disease studies
NICE: National Institute for Health and Care Excellence
REPRISE: Replicating Evidence of Preserved Renal Function an Investigation of Tolvaptan Safety Efficacy in ADPKD trial
RRT: Renal replacement therapy
TEMPO: Tolvaptan Efficacy and Safety in Management of Autosomal Dominant Polycystic Kidney Disease and its Outcomes study
THIN: The Health Improvement Network
TKV: Total kidney volume
